# Chewing lice (Phthiraptera) on a wild Golden eagle (*Aquila chrysaetos*) and a zoo-kept Eurasian griffon vulture (*Gyps fulvus*) in Tyrol, Austria

**DOI:** 10.1007/s00436-025-08531-y

**Published:** 2025-07-21

**Authors:** Jutta Pikalo, Oldrich Sychra, Miguel Peña-Espinoza, Maryna Galat, Maria Sophia Unterköfler, Mike Heddergott, Walter Glawischnig, Hans-Peter Fuehrer

**Affiliations:** 1 Institute of Parasitology, University of Veterinary Medicine Vienna, Veterinärplatz 1, 1210 Vienna, Austria; 2 Department of Biology and Wildlife Diseases, Faculty of Veterinary Hygiene and Ecology, University of Veterinary Sciences Brno, Brno, Czech Republic; 3 Institute of Parasitology, Department of Infectious Diseases and Pathobiology, Vetsuisse Faculty, University of Bern, Länggassstrasse 122, 3001 Bern, Switzerland; 4 Faculty of Veterinary Medicine, National University of Life and Environmental Sciences of Ukraine, Heroiv Oborony Str. 15, Kiev, 03041 Ukraine; 5 Department of Zoology, Musée National d Historire Naturelle, 2160 Luxembourg, Luxembourg; 6 Institute for Veterinary Disease Control Innsbruck, Austrian Agency for Health and Food Safety Ltd. (AGES), Technikerstrasse 70, 6020 Innsbruck, Austria

**Keywords:** Chewing lice, *Colpocephalum turbinatum*, *Craspedorrhynchus aquilinus*, *Falcolipeurus quadripustulatus*, *Aquila chrysaetos*, *Gyps fulvus*

## Abstract

Chewing lice (Phthiraptera) are obligate and permanent ectoparasites commonly found on birds. The life cycle of these insects is completed on the body of the host and therefore many are host specific. This is the first report of chewing lice on a wild Golden eagle (*Aquila chrysaetos*) and a zoo-kept Eurasian griffon vulture (*Gyps fulvus*) in Tyrol, Austria. Three different species of chewing lice were identified: *Craspedorrhynchus aquilinus* was found on *Aquila chrysaetos* and *Colpocephalum turbinatum* and *Falcolipeurus quadripustulatus* were found on *Gyps fulvus*. The lice were identified morphologically and by barcoding. Chewing lice (Phthiraptera) of eagles, vultures, and other Accipitriformes are understudied, and further research is needed.

## Introduction

Chewing lice (Phthiraptera) are permanent, obligate and often host-specific ectoparasites in birds (Price et al. 2003). They are small (from ≤ 1 mm to about 10 mm in length), wingless, dorsoventrally compressed insects that parasitize birds and some mammals (Clayton et al. 2008). More than 300 genera and 5,300 species of parasitic lice in five parvorder are known: Amblycera, Ischnocera, Trichodectera, Rhynchophthirina, and Anoplura (Pérez 2023; Galloway 2018). Approximately 4,000 species of bird lice have been described worldwide (Gustafsson et al. 2024).

Lice are typically transmitted during close contact between individuals (Talabante and Bernal 2022). In general, chewing lice are relatively benign parasites, but if they occur in large numbers on a bird, they can cause severe pruritus, small holes on feathers, plumage quality decay, reduced feather mass, and an increase of feather breakage, leading to changes in flight performance, body mass, thermoregulation, survival, and sexual selection of the hosts (Price et al. 2003; Clayton et al. 2008). Beside their direct pathogenicity, they can also act as vectors for bacteria, fungi, and filarioid nematodes (Martín-Mateo 2002).

The Golden eagle, *Aquila chrysaetos* (Linnaeus 1758), is a large raptor which is found in North America, Europe, northern Africa, and Asia (Katzner et al. 2020). As a monogamous, and long-lived species, the Golden eagle is selected for uniform and long-term use of its habitat (Landmann and Mayrhofer 2001). Habitat alteration, especially urbanization and human population, has important, often negative effects on the Golden eagle. There are reports about mortality caused by wind turbines and power lines (Katzner et al. 2020; Kagan 2016).

The Eurasian griffon vulture, *Gyps fulvus* (Hablizl 1783), is a large vulture feeding primarily on the carcasses of domestic and wild ungulates. As a long-lived monogamous species, living in small colonies in many parts of Europe, threats to its survival across its whole range include poisoning, collision with wind turbines and power lines, electrocution, and illegal killing. The Eurasian griffon vulture is protected in numerous countries and breeding and there have been several reintroduction programs (Salvador 2024).

Eight species of chewing lice have been documented to infest Golden eagle: *Colpocephalum flavescens* (De Haan 1829), *Colpocephalum impressum* Rudow, 1866, *Craspedorrhynchus aquilinus* (Denny 1842), *Degeeriella aquilarum* Eichler 1943, *Degeeriella fulva* (Giebel 1874), *Falcolipeurus suturalis* (Rudow 1869), *Laemobothrion maximum* (Scopoli 1763), *Laemobothrion vulturis* (Fabricius 1775) (Price et al. 2003).

Seven species of chewing lice are known to infest Eurasian griffon vulture: *Aegypoecus trigonoceps* (Giebel 1874), *Colpocephalum gypsi* (Eichler and Złotorzycka 1971), *Colpocephalum turbinatum* Denny 1842, *Cuculiphilus* (*Aegypiphilus*) *gypsis* (Eichler 1944), *Falcolipeurus quadripustulatus* (Burmeister 1838), *Laemobothrion vulturis* (Fabricius 1775), *Nosopon casteli* Tendeiro 1959 (Price et al. 2003).

Genera of chewing lice are typically restricted to one avian host family or order, but *Colpocephalum* spp. occur on 11 distantly related avian host orders (Catanach et al. 2018).

Kutzer et al. (1980) conducted investigations into the parasitic fauna associated with raptors in Austria. However, Golden eagle and Eurasian griffon vulture were not included in their examination. Although chewing lice were recorded, the species identified differed from those reported in the present study. Consequently, there is no data on the occurrence of chewing lice associated with Golden eagle or Eurasian griffon vulture in Austria. Here, we present a case report of the occurrence and molecular characterisation of chewing lice on a Golden eagle and a Eurasian griffon vulture in Tyrol, Austria.

## Material and methods

In March 2022, an electrocuted, adult (> 5 years old, 3.4 kg) male Golden eagle was found by a ski tourist in Tyrol, Austria. The bird was brought to the Institute for Veterinary Disease Control Innsbruck at the Austrian Agency for Health and Food Safety (AGES), for routine pathological analysis. The bird had a good nutritional status and a poor maintenance status. During the necropsy, lice were observed between head and neck feathers, and some were preserved in 70% ethanol. The lice were sent to the Institute of Parasitology, University of Veterinary Medicine Vienna (Vetmeduni Vienna), for identification and molecular barcoding.

In June 2023, an adult (around 50 years old, 5.5 kg) male Eurasian griffon vulture kept at the Alpenzoo Innsbruck died and was sent to the Institute for Veterinary Disease Control Innsbruck (AGES) for necropsy. This bird originally came from Spain and was brought to the Alpenzoo Innsbruck in 2019 with an interim stay in Italy from 1992 to 2019. At the Alpenzoo, it was kept in an aviary with other European griffon vultures, alpine crows and Egyptian vultures (*Neophron percnopterus* (Linnaeus 1758)). Nutritional and maintenance status of this bird were poor. Some of the found lice were preserved in 70% ethanol and sent to the Institute of Parasitology at the Vetmeduni Vienna for species identification.

### Morphological and molecular specification

Lice were mounted on slides for morphological characterisation and identified using the keys by Gallego et al. (1987) for *Craspedorrhynchus,* Price and Beer (1963) for *Colpocephalum* and Złotorzycka (1980) for *Falcolipeurus*, and analysed with a stereo microscope (Nikon SMZ1270, Tokyo, Japan) prior to molecular barcoding.

For molecular analysis, one leg of individual lice was subjected to total DNA extraction using the DNEasy Blood & Tissue Kit (Qiagen, Hilden, Germany) under conditions described previously (Peña-Espinoza et al. 2023). Selected specimens (10 lice collected from the Golden eagle and 14 from the Eurasian griffon vulture) were individually subjected to DNA barcoding analysis. A conventional PCR targeting the insects'mitochondrial cytochrome oxidase subunit 1 (*COI*) gene was used (Primers: Lep-F1/Lep-R1; Hebert et al. 2004) under conditions previously described (Peña-Espinoza et al. 2023). PCR products were sequenced at LGC Genomics GmbH (Berlin, Germany). The resulting mt *COI* sequences were compared with databases using BOLD (http://www.boldsystems.org/) and Blast (https://blast.ncbi.nlm.nih.gov/Blast.cgi).

## Results

### Morphological and genetic identification

**Fig. 1:**
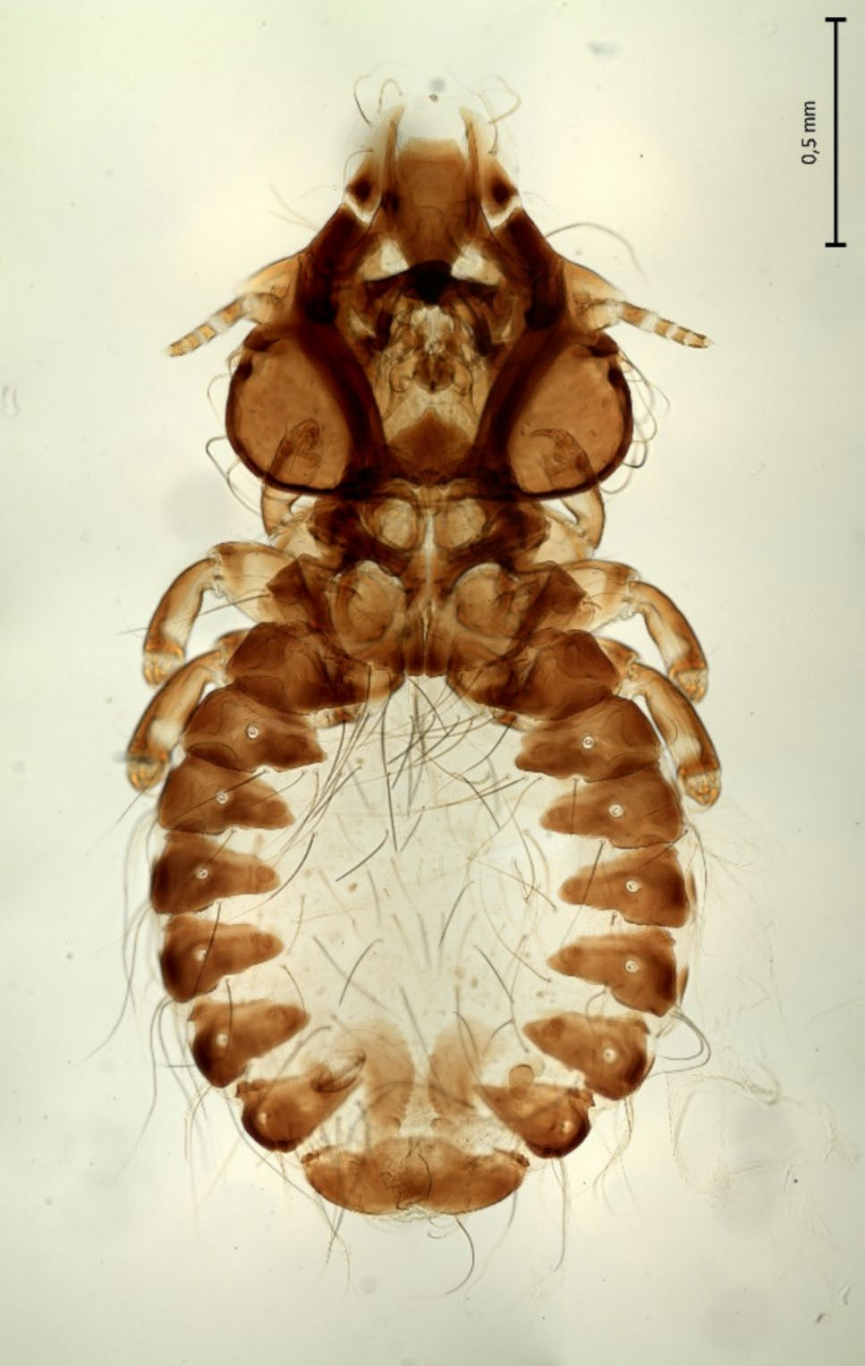
Female of *Craspedorrhynchus aquilinus*

**Fig. 2:**
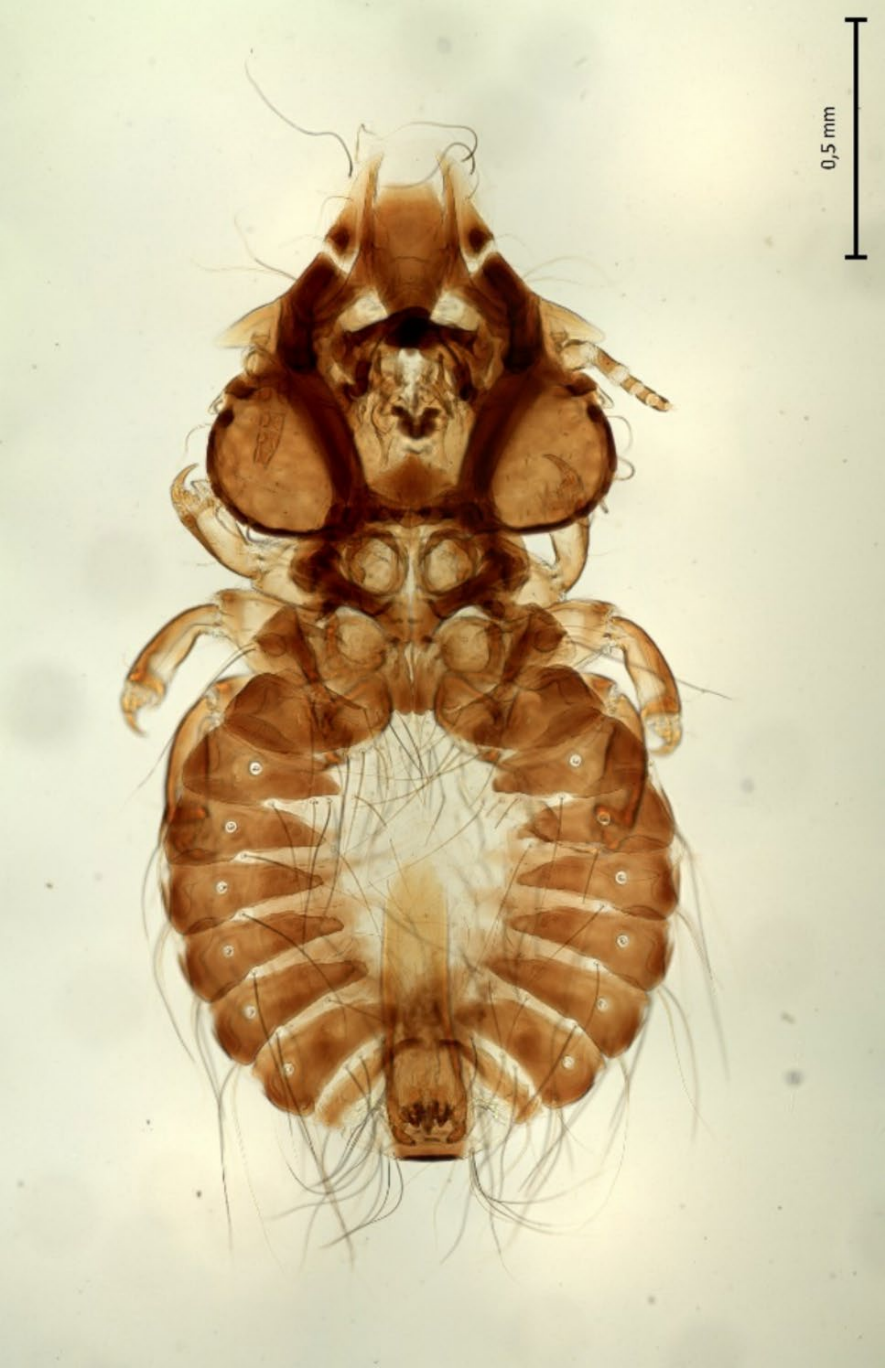
Male of *Craspedorrhynchus aquilinus*

All ten chewing lice collected from the wild Golden eagle were adult *Craspedorrhynchus aquilinus* by morphological analysis (Figs. 1 and 2).

Our sequences obtained from *C. aquilinus* (GenBank: OR732377, OR732374, OR732380 and BOLD: PAVEA_SAF8-PAVEA_SAF10) show 75% identity to a sequence from *C*. *haematopus* (MZ660348) collected in Finland (Roslin et al. 2022).

**Fig. 3:**
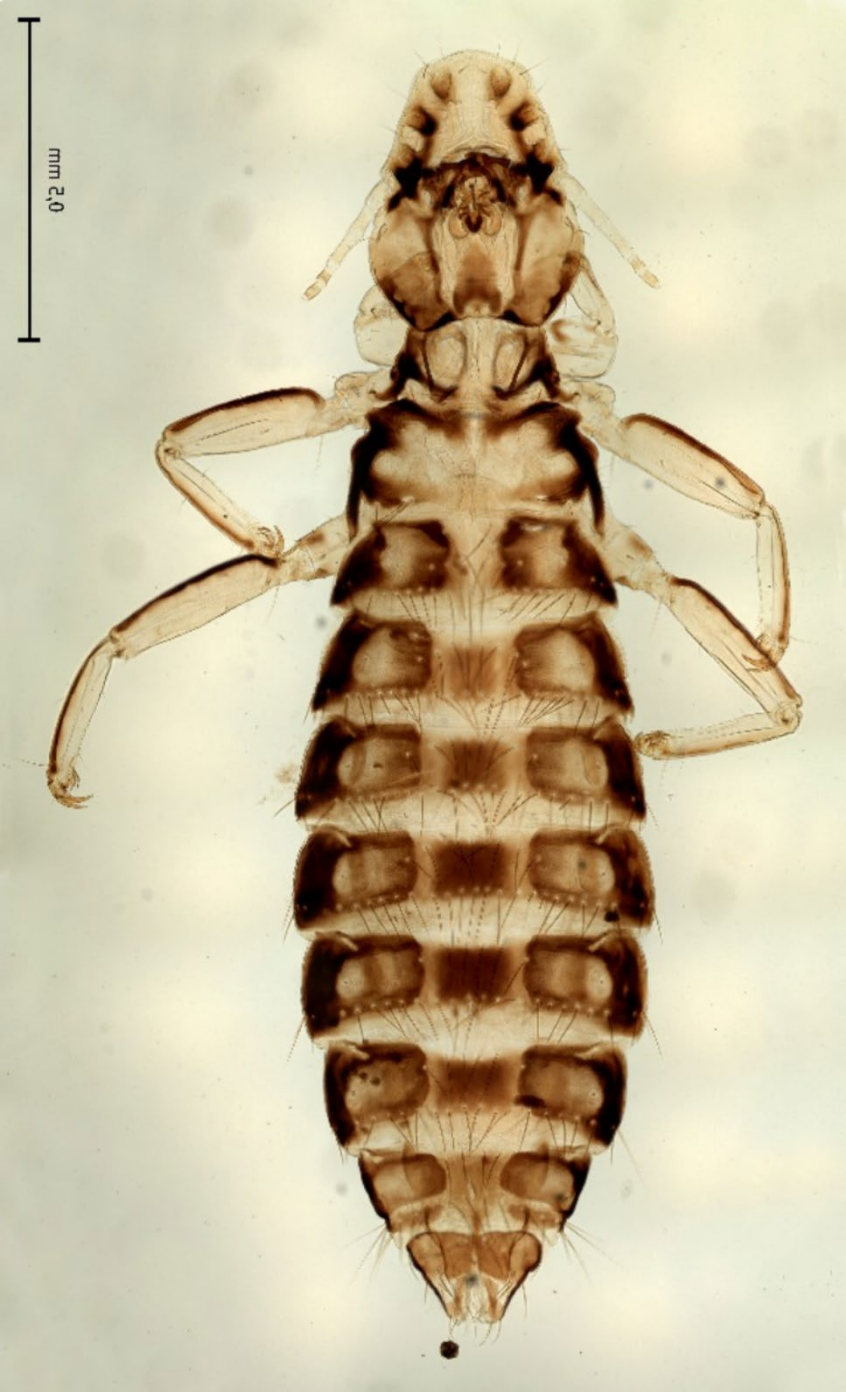
Female of *Falcolipeurus quadripustulatus*

**Fig. 4:**
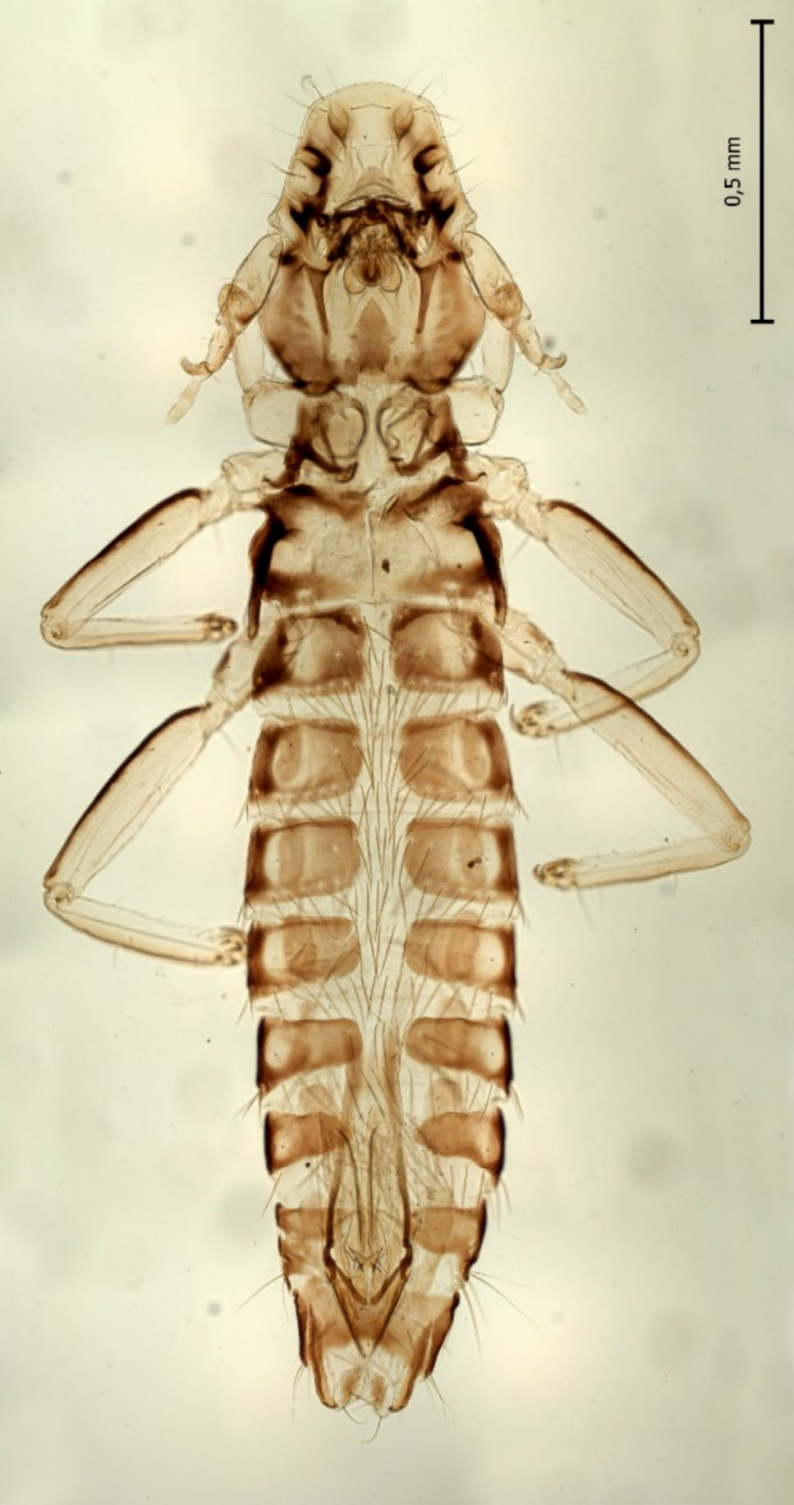
Male of *Falcolipeurus quadripustulatus*

**Fig. 5:**
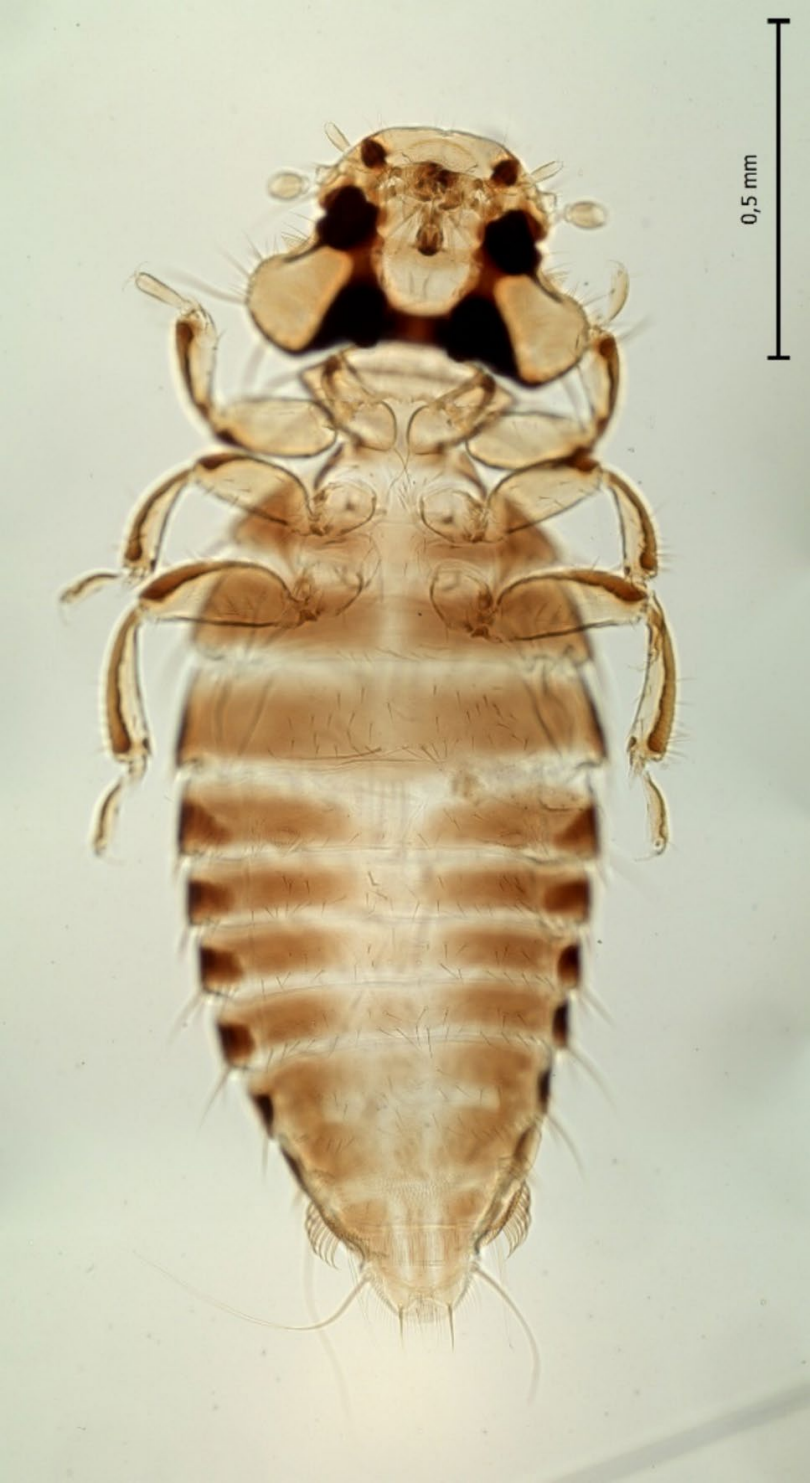
Female of *Colpocephalum turbinatum*

Of the 14 analysed adult chewing lice two species were identified in the zoo-kept Eurasian griffon vulture based on morphological characteristics: *Falcolipeurus quadripustulatus* (Figs. 3 and 4) and *Colpocephalum turbinatum* (Fig. 5).

Our sequences obtained from *F. quadripustulatus* (BOLD: PAVEA-F1, PAVEA-F2, and PAVEA-F4; GenBank: OR732376, OR732378, OR732379) were 99.7% identical to a *F. quadripustulatus* sequence from China (MH001226; Song et al. 2019).

The sequences for* C. turbinatum* (BOLD: PAVEA_GF; GenBank: OR732375) were 100% identical to lice named as *C*. *griffoneae* Ansari 1955 from a vulture from China (NC039530 and MH001228; Song et al. 2019) and 96% identity to *C*. *turbinatum* from a *Bubo lacteus* from Malawi (MF443990; Catanach et al. 2018).

## Discussion

*Craspedorrhynchus aquilinus* is monoxenous, infesting the Golden eagle (Price et al. 2003). To our knowledge, *C. aquilinus* has been reported in several countries, including: Czechia (Balát 1977), Finland (Hackman 1994), France (Seguy 1944), Germany (Mey 2004), Hungary (Vas et al. 2012), Italy (Manilla 2000), Romania (Adam 2008), Slovakia (Ošlejšková et al. 2021), Spain (Martin-Mateo (Martín-Mateo 1989); Pérez et al. 1996), Sweden (Gustafsson et al. 2018), Turkey (Eren et al. 2022), Iran (Bahiraei et al. 2024), and the USA (Heckmann 2014). However, only Pérez et al. (1996) reported a prevalence of 33.3% for *C. aquilinus* on *A*. *chrysaetos* but derived from looking at only three birds.

There are few sequences within *COI* of this genus, with no available sequence of *C*. *aquilinus*. Our *C*. *aquilinus* sequences (GenBank: OR732377, OR732374, OR732380 and BOLD: PAVEA_SAF8-PAVEA_SAF10) are the first to be uploaded in gene banks, and with 75% identity are closest to a sequence from *C*. *haematopus* isolated in Finland (MZ660348; Roslin et al. 2022). More sequences are needed for the genetic characterisation of *C*. *aquilinus*.

In the zoo-kept Eurasian griffon vulture, there were two different chewing lice species identified based on morphological characteristics, *F*. *quadripustulatus* and *C*. *turbinatum*. Talabante and Bernal (2022) also collected *F*. *quadripustulatus* and *C*. *turbinatum,* but in a wild breeding colony of Eurasian griffon vultures in central Spain.

*Falcolipeurus quadripustulatus* has been previously reported to infest Eurasian griffon vultures, as well as seven other hosts (Price et al. 2003): three African species, Cape vulture, *Gyps coprotheres* (Forster 1798), Rüppell’s vulture, *Gyps rueppelli* (Brehm 1852), Nubian vulture, *Torgos tracheliotos* (Forster 1796) (Ledger 1980), two species of vultures in the Oriental Realm, Himalayan vulture *Gyps himalayensis* (Hume 1869), and Indian vulture *Gyps indicus* (Scopoli 1786) (Tandan 1964; Lakshminarayana 1979; Nimsuphan et al. 2011), Cinereous vulture, *Aegypius monachus* (Linnaeus 1766) in the Palearctic Realm: Czechia (Balát 1977), Bulgaria (Ilieva 2009), Romania (Adam 2008), Turkey (Dik et al. 2013, 2022), and India (Lakshminarayana 1979) and from Short-toed snake eagle, *Circaetus gallicus* (Gmelin 1788), for example, from Turkey (Dik et al. 2022). Occurrence of *F. quadripustulatus* on Eurasian griffon vultures has been reported from Bulgaria (Ilieva 2009), Italy (Manilla, 2000), Germany (Mey 2004), Poland (Złotorzycka 1980), Portugal (Tomás et al. 2016), Romania (Adam 2008), Spain (Martin-Mateo 1989); Talabante and Bernal 2022), India (Lakshminarayana 1979), and Iran (Bahiraei et al. 2024).

Talabante and Bernal (2022) reported a prevalence of *F. quadripustulatus* of 61% in a wild breeding colony of vultures (*n* = 28) in Spain, which is consistent with an earlier study by Pérez et al. (1996), who reported a 52.9% prevalence in *G*. *fulvus* in the same region. In contrast Tomás et al. (2016) found 6 of 6 examined Eurasian griffon vultures infested, with mean intensity of 2.17 of *F. quadripustulatus*. Mey et al. (2016) examined nestlings (n = 7) of Black vultures, *Aegypius monachus* (Linnaeus 1766), from Mongolia with a prevalence of 100% and mean intensity of 4.2. This is consistent with the findings of Pérez et al. (1996), who also reported 100% infestation of *A*. *monachus* in Spain.

*Colpocephalum turbinatum* according to morphological characterisation, but our *Colpocephalum* sequences revealed 100% identity to lice named as *C*. *griffoneae* from China (NC039530 and MH001228; Song et al. 2019) and 96% identity to *C*. *turbinatum* (MF443990; Catanach et al. 2018). However, Song et al. (2019) only mention that the louse was from a vulture and only genetic analysis without morphological examination was performed.

*Colpocephalum griffoneae* was described by Ansari (1955) from a Himalayan vulture. Price and Beer (1963) wrote in their review: „We have not seen the supposedly „first “ description of *C. griffoneae*. However, the „second “ and „third “ descriptions offer few identification features. The illustrations, when present, are poor and the written description meaningless. Whether or not this may be *C. turbinatum* we cannot tell. It is best for the present to make no guess as to its identity.“ So they placed this species as „species sedis incertae “. Contrary to Price and Beer (1963), we were able to see the „first “ description by Ansari (1955), but it is also poor without any drawings. To our knowledge, there is no adequate description of this louse, so it is unfortunately impossible to identify it. Moreover, Naz et al. (2020) wrote that type material is listed as deposited in the NHML by Ansari (1955), but it could not be found and is presumed lost. For this reason, Nimsuphan et al. (2011), who collected *Colpocephalum* from Himalayan vultures in Thailand, identified these lice only to genus level as “*Colpocephalum* sp.”. Contrary to these authors, Song et al. (2019) mention *C. griffoneae* from Himalayan vultures in their paper. Unfortunately, there is no description regarding how Song et al. (2019) identified their specimens. Since it is not possible to distinguish *C. griffoneae* morphologically from *C. turbinatum*, there is no doubt that “identification” of “*C. griffoneae*” was based solely on the basis of host association. Despite the fact that the sequence of our *C. turbinatum* sample is identical with those of “*C. griffoneae*”, it also shows only 4% difference from other *C. turbinatum* sequences. Such a small sequence diversity falls well within the intraspecific variability of the *COI* gene in lice. For example, Kolencik et al. (2017) proposed that a clear and reliable limit for intraspecific variability of the *COI* gene in amblyceran lice could be at 12% divergence. Thus, we assume that lice named “*C. griffoneae*” by Song et al. (2019) in fact represent *C. turbinatum*.

*Colpocephalum turbinatum* is a euryxenous louse species with 52 hosts from 4 orders (Accipitriformes 42 hosts, Strigiformes 5 hosts, Falconiformes 2 hosts, and Columbiformes 3 hosts) (Price et al. 2003).

The following members of the genus *Gyps* have been reported as hosts for *C*. *turbinatum*: Eurasian griffon vulture, Egyptain vulture, Cape vulture, white-rumped vulture, *G*. *bengalensis* (Gmelin 1788), white-backed vulture, *G*. *africanus* (Salvadori 1865), Indian vulture, and Rüppell's vulture. Moreover, *C. turbinatum* has been documented infesting several Accipitriformes present in Austria, including common Eurasian buzzard, *Buteo buteo* (Linnaeus 1758), Western marsh harrier, *Circus aeruginosus* (Linnaeus 1758), Black kite *Milvus migrans* (Boddaert 1783), and Barn owl *Tyto alba* (Scopoli 1769) (Adly et al. 2022; Price et al. 2003).

*Colpocephalum turbinatum* has an almost cosmopolitan distribution. In Europe, this louse has been reported from Bulgaria (Ilieva 2009), Czechia (Balát 1977), Finland (Hackman 1994), France (Seguy 1944), Germany (Mey 2004), Hungary (Vas et al. 2012), Italy (Manilla 2000), Poland (Złotorzycka 1976), Portugal (Tomás et al. 2016), Romania (Adam 2008), Slovakia (Ošlejšková et al. 2021), Spain (Martin-Mateo 1989); Talabante and Bernal 2022), Sweden (Gustafsson et al. 2018), and Turkey (Eren et al. 2022).

In detail, the occurrence of *C. turbinatum* in *Gyps fulvus* has been documented in Portugal, with a prevalence of 50% (*n* = 6) and a mean intensity of 1.67 (Tomás et al. 2016). In Spain, Talabante and Bernal (2022) reported a prevalence of 46% in a wild breeding colony of *G. fulvus* (*n* = 28), while Pérez et al. (1996) found a prevalence of 52.9% in wild-caught individuals of the same species. Furthermore, *C*. *turbinatum* has been reported to infest White-rumped vulture and Indian vulture in India (Lakshminarayana 1979), White-backed vulture from Bechuanaland (now Botswana), Cape vulture from Transvaal (now South Africa), and Rüppell's vulture from Somaliland (now: Somalia) (Price and Beer 1963).

The infection of a zoo-kept Eurasian griffon vulture with two species of chewing lice having a broad host-spectrum leads to the assumption that the animal possibly had contact with wild animals, or that there is a stable population of the parasites within the zoo-kept vulture population. *Colpocephalum turbinatum* has also been documented in the Egyptian vulture (Adly et al. 2022). Given that this species was housed in proximity to the Eurasian griffon vulture, the cohabitation within the zoo environment represents a plausible source of infestation. If it was a captured wild bird, the chewing lice may have survived and reproduced on their host in captivity for many years (Naz et al. 2024; Gustafsson et al. 2021). If there are small populations of chewing lice they can be easily overlooked, especially on a large bird such as a vulture. Lice that are host specific usually cannot colonize hosts of different species in the same aviary or zoo. On the other hand, it is good to clinical examine the birds, especially in the case of stenoxenous or euryxenous species such as *F*. *quadripustulatus* and *C*. *turbinatum*. The increasing intensity of grooming can also indicate an infestation with ectoparasites which can be seen by animal-caretakers (Bush et al. 2023). However, even it is useful to check birds for chewing lice and also for eggs in primary feathers, there is generally no reason to treat the bird prophylactic or eradicate the lice (or other ectodes), especially in endangered birds if the birds are in a good condition. Due to the lack of data on chewing lice in raptors which are kept in the zoos we suggest to explore the dynamics and host-parasite interactions in further studies. Knowledge of the ectoparasites associated with captive bird species is also important for zoos, particularly in the context of implementing effective hygiene and biosecurity measures. The degree to which climatic variables affect the prevalence and intensity of ectoparasite infestations in avian hosts remains to be elucidated in the respective geographical region (Frixone et al. 2025).

Chewing lice (Mallophaga) of accipitriform birds (including eagles and vultures) are understudied and neglected parasites and information about the presence of these parasites and barcoding sequences in databases are scarce. Within this study, chewing lice collected on a wild Golden eagle and a zoo-kept Eurasian griffon vulture were analysed and confirmed as *C*. *aquilinus* in the Golden eagle and *F. quadripustulatus* and *C. turbinatum* in the Eurasian griffon vulture. Additional studies are needed to obtain an insight into the species diversity of chewing lice in wildlife, not only in Austria, but elsewhere.

## Data Availability

No datasets were generated or analysed during the current study.
